# Customer Engagement Around Cultural and Creative Products: The Role of Social Identity

**DOI:** 10.3389/fpsyg.2022.874851

**Published:** 2022-04-25

**Authors:** Zaiyu Zhang, Wenjia Li

**Affiliations:** College of Communication and Art Design, University of Shanghai for Science and Technology, Shanghai, China

**Keywords:** social identity, cultural and creative products, customer engagement behaviors, social media, purchase intention

## Abstract

Along with the increasing trend of transactions occurring on social media, the consumption of Chinese cultural and creative products has increased even against the background of the COVID-19 pandemic. In this context, this article aims to analyze the relationships between virtual community-based social identity and cultural and creative product customer engagement (CE) behaviors. To this end, social identity theory and CE behavior theory were applied to previous research model. Structural equation modeling (SEM) was conducted using data from 520 self-administered questionnaires from online virtual community members. The results show that social identity has a significant effect on customer knowledge behavior, participation behavior, and influencer behavior. Moreover, influencer behavior mediates the effect of social identity on purchase intention. The study also identified gender differences in the mediation for influencer behaviors. Our results suggest that women are more sensitive to influencer behaviors than men, and thus generate more purchase behaviors.

## Introduction

According to the Chinese National Bureau of Statistics (2021), the business revenue of enterprises in China’s cultural and related industries increased by 2.2% in 2020 compared to the previous year. The museum industry grew rapidly in China between 2011 and 2021 (Chinese National Bureau of Statistics, 2021). The last 2 years have presented many obstacles to museum visits due to the COVID-19 pandemic. However, the statistics mentioned above show that the museum industry still maintained a relatively high growth rate during the pandemic. A typical example of these successes is one of the cultural products developed by Henan Museum in 2020. This was in the form of a blind box with characteristics resembling those of museum artifacts, and by digging themselves, customers could find and open the blind box to reveal scaled-down versions of the artifacts, allowing them to experience the fun of digging for treasure. However, in 2020, people’s enthusiasm for visiting the exhibition remained relatively low due to the social isolation measures brought about by the pandemic. So how did this museum creation generate such a buzz under such difficult circumstances? Through our investigation, we found that the popularity of the Henan Museum’s product was due to enthusiasts sharing posts about it on social media, which triggered even more attention. The search term #henanmuseumarchaeologyblindbox was viewed more than 28 million times on Weibo—the largest social media platform in China—and discussed more than 12,000 times in less than a month.

Inspired by this phenomenon, we assumed that interest-based online communities must also contribute to brand growth in a subtle way; we wanted to find out how the sharing behavior of these enthusiasts on social media, which caused other enthusiasts to purchase the product, actually occurred. How can brands leverage interest-based communities to help their brands grow?

Interest-based communities are communication platforms created spontaneously and autonomously by certain users, while other users search and join these interest-based platforms on their own initiative. There are many communities of fans of cultural and creative products on Chinese Internet platforms, such as the “We All Love to Buy Cultural and Creative Products” group on Douban where a group of people who are interested in museum cultural products gather. These users actively exchange and share their purchase experiences and their knowledge about certain products, and also disseminate knowledge and perceptions of new products rapidly in these communities. In the community, the topics discussed inevitably include the attributes of certain products, which means that every discussion about cultural and creative products is, for a brand, an act of consumer participation in the brand. At this level, community members are co-creators of brand value. Through online interaction, people can get the information they need and buy the products they are interested in without having to leave home. As for minor culture, virtual communities help promote brand familiarity. Interest-based members are able to form social relationship nets more easily (Zhao et al., 2009), which also provides potential opportunities for increasing customer engagement (CE) behaviors. CE through social media generates positive impressions of the brand (Osei-Frimpong and McLean, 2018).

Previous studies have focused on the impact on consumer behavior of brand communities created by firms for the purpose of binary interactions with consumers (Zaglia, 2013; Elbedweihy et al., 2016). He et al. (2012) argued that identification with the company on the part of consumers causes engagement behaviors. Researchers have also explored various theories to explain community member behaviors and to examine the relationship between community identity and community participation behavior in online Internet communities. The engagement behaviors in these interest-based virtual communities imply differences in the social psychologies of their users. Social identity is considered to be a motivation to participate in online communities. Bagozzi and Dholakia (2002) study adopted a marketing viewpoint to identify how social influence and variable social identity impact virtual community participation. The focus of the present study is on how social identity influences engagement behaviors with brands among members of user-created interest-based communities. This is valuable for the study of brand communities, because the spontaneity of user-created community members differs from the passivity of members of brand communities: in user-created interest-based communities members’ behavior is autonomous rather than passive. Another difference between user-created communities and brand communities is that the content exchanged by members of user-created communities may include experiences of a large number of different brands, rather than a single brand. It is also valuable for brands to gain insight into the psychology of consumers and trends in content production within the community from the perspective of consumer self-motivation, as a complement to the brand-consumer relationship, and this can provide a consumer perspective on the direction of product marketing.

This study will examine the relationship between community identity and the within-community behavior of members of a Chinese Internet community which is based on interest in cultural and creative products. To determine how social influences impact users’ engagement, our model uses social identity theory framing. Once the role of social identity in user-created communities is established, subsequent studies can continue to further explore the motivation of social identity.

## Literature Review and Hypothesis Development

### Social Identity Theory

Social identity has been defined by Tajfel (1978) as “that part of an individual’s self-concept which derives from his knowledge of his membership of a group (or groups) together with the value and emotional significance attached to the membership.” Tajfel (1978) research on categorization and the development of accentuation theory highlighted the cognitive consequences of (social) categorization processes, an important component of social identity theory and later of self-categorization theory. He proposed that classifying physical stimuli could lead people to perceive between-category differences as larger (Tajfel and Wilkes, 1963). Tajfel (1978) defined social identity (or group identity), in both cognitive and evaluative terms, as that part of the self-concept corresponding to the knowledge of group membership, together with the value and emotional significance of that membership. This has proven to further influence different fields such as organizational behavior (Ashforth and Mael, 1989) and fandom behavior study (He, 2005). This paper argues that social identity theory can be used to study the influence of individual psychological characteristics on the behavior of community members who share similar passions for cultural and creative products.

Tajfel’s definition of social identity involved cognitive, affective, and evaluative components (Bergami and Bagozzi, 2000). In a cognitive sense, social identity is a self-categorization process whereby the individual forms a self-awareness of virtual community membership, which includes awareness of both similarities with other members and dissimilarities with non-members (Ashforth and Mael, 1989). In this case, the members of the community share a similar passion for cultural and creative products. Tajfel (1978) definition also implies that a virtual community has an emotional and evaluative impact on its members. In the emotional dimension, social identity represents a sense of emotional investment in the group, which researchers have defined as attachment or affective commitment (Bagozzi and Dholakia, 2002). Affective social identity strengthens members’ bonds with each other and fosters their engagement behaviors in the group (Ellemers et al., 1999; Bergami and Bagozzi, 2000). Aside from affective social identity, evaluative components also play a crucial role in explaining users’ coherence in a group (Bergami and Bagozzi, 2000). The evaluative component represents group self-esteem and is defined as the evaluation of self-worth based on community “belongingness” (Ellemers et al., 1999). Members of the cultural and creative community, the subject of this paper, develop their contributions to the community based on their perceptions of cultural and creative preferences. The emotion of investment also leads them to identify with, and emotionally link themselves to, the community.

Despite the discrepancies among these three dimensions, they have not been fully studied in the current literature; instead, social identity has generally been measured and treated as a unidimensional construct (Ellemers et al., 1999). In this study, we propose that each dimension exerts a positive effect on CE behaviors, and that the cognitive, affective, and evaluative dimensions are first-order construct components of a second-order social identity construct.

### Customer Engagement Behavior

The concept of CE emerged in 2006 as a sub-set of the term “engagement” related to the study of customers’ behaviors and emotions in terms of their interaction/participation with brands (Vivek et al., 2014). According to the literature, most CE-related studies have viewed CE from a behavioral perspective, considering that engagement behaviors—especially non-transactional behaviors—can affect a company’s development (Verhoef et al., 2010). Non-transactional behaviors indirectly produce profits for a firm (Kahn, 1990). Furthermore, CE behavior has been examined beyond purchase behavior; this research was driven by the understanding that a consumer’s motivation comes from the emotional bond between them and a company (Van Doorn et al., 2010). Prior literature has considered non-transactional behaviors to be comprised of various related behaviors such as word-of-mouth (WOM) information transmission, referrals, co-creations, and ratings (Van Doorn et al., 2010; Wei et al., 2013). In the online community, users’ spontaneous exchanges of opinions and sharing of knowledge about a product represent the emotional ties between users and the product, which are related to CE behaviors. At the same time, the extensive discussions about products and brands generated by customers’ engagement in the community stimulate the interest of other members within the community in these brands. As a result, when consumers instinctively identify with a brand, positive feedback is generated accordingly.

The creation of value by customers for firms occurs through a more elaborate mechanism than purchase alone (Kumar et al., 2010). In their study of customer engagement value (CEV), Kumar et al. (2010) proposed four components of CEV: customer life value, customer referral value, customer influencer value, and customer knowledge value. These four dimensions of CEV can be derived from four dimensions of customer behavior: (1) customer purchase behavior, (2) customer referral behavior, (3) customer influencer behavior, and (4) customer knowledge behavior. The four dimensions of customer behavior proposed by Kumar et al. (2010) are based on the combination of the concept of purchase behavior with the three other defined behaviors. Customer referral behavior is extrinsically motivated by, and related to, the acquisition of new customers. Almost all behavior that would be classified as contributing to consumer influencer behavior is based on the intrinsic motivation of the customer. Customer influencer behavior is mostly represented by WOM activity, such that a customer voluntarily generating WOM about the firm and its products is engaging in consumer influencer behavior. Customer knowledge behavior includes feedback behaviors, such as providing feedback to a community or firm. In the process of developing knowledge, customers assist a company by adding value (Joshi and Sharma, 2004).

Based on this CEV theory, we split CE behavior into four similar dimensions—in this case, four outcomes of social identity. Customer influencer behavior is the perceived behavior of community members as influenced by others. Customer participation behavior is an indicator of how active the community is for individual users. Knowledge behavior is the interaction and information sharing that occurs between customers about a product.

### Research Model and Hypotheses

**FIGURE 1:**
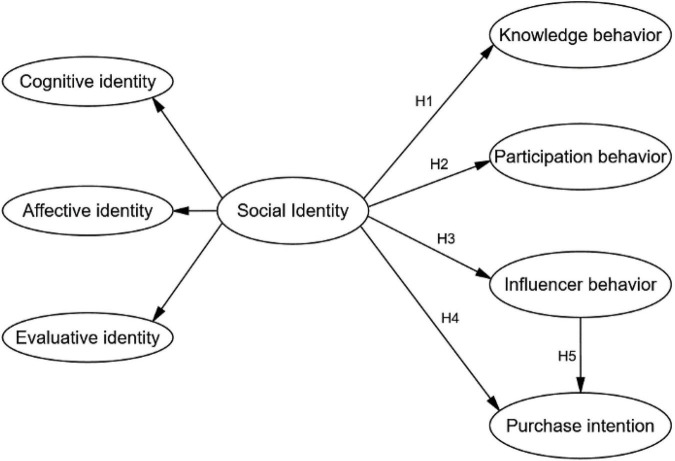
The research model.

Zhou and Lu (2009) have proposed a model to explain the correlation between social identity, community members’ sharing intentions, and their actual behavior. This model specifically considers members’ knowledge-sharing behavior. We extracted part of this model and added other customer behaviors as outcomes to adapt the model to our research, using the social identity theory (Tajfel, 1978) and consumer engagement behavior theory as underlying frameworks (Figure 1).

The correlation between social identity and CE has been widely studied. Social factors have been commonly used as mediators or antecedents in various CE behavior studies (Shen, 2012; Sharma and Crossler, 2014; De Oliveira et al., 2016). A previous study has also verified the importance of social presence to the extent that it has been found to impact customers’ sociable interactions in the social commerce (s-commerce) environment (Xiang et al., 2014). Other studies have examined the mediating effect of social factors on the behavior of online customers participating in s-commerce activities (Hajli et al., 2015; Kucharska, 2019). Prior research on the overall effect of these three dimensions on social identity has generally supported positive associations with CE behaviors, such as participation behaviors. As a minor cultural group, cultural and creative product consumers, and the degree to which their behavior is affected by their group identity, represent the starting point for this study. Prior literature about social media’s role for firms has been more focused on researching brands’ social media pages. Past studies have revealed how social interaction motives influence CE (de Silva, 2019). In particular, in an online community context, the ways in which customers’ identification with their community might increase CE behavior have also been studied (Prentice et al., 2019). This study explores how interest-based virtual communities are also able to generate a positive impact on customers’ engagement behaviors. Moreover, with the knowledge that existing consumer behavior studies have focused more on specific behaviors, such as participation behavior (Dholakia et al., 2004) or knowledge behavior (Shi, 2010), this study examines four components of CE behavior to more generally identify the influence of social identity.

Past studies have supported the position that searching for information is the main form of gratification that consumers seek in online brand communities (Zaglia, 2013). When customers identify with companies, they are more likely to develop a psychological attachment to them and to voluntarily contribute to the company’s goals (Bhattacharya and Sen, 2003). Posting and sharing information is one of the ways in which community members can actively develop a mental attachment to a company. Based on the above discussion, we have developed the following hypotheses:

Hypothesis 1: Social identity positively influences knowledge behavior in cultural and creative product virtual communities.

Most studies use social motivations to explain active participation in virtual communities (Tsai and Bagozzi, 2014; Chih et al., 2017; Hsu et al., 2018). Social identity is one of the social motivations for virtual community participation (Bagozzi and Dholakia, 2002; Hsu et al., 2018), which develops through in-group ties prompting people to interact with other members (Wolter and Cronin, <suffix>Jr.</suffix>, 2017). Researchers believe that a user’s social identification with social media means that the user has a sense of belonging to that social media community and will willingly continue to engage with the social media platform (Yang et al., 2021). Members who are connected to the community are more likely to dedicate more time and energy to engaging in community activities (Choi, 2013). We assumed that social identity played a similar role in members’ participation behavior in the user-created interest community. On the basis of the this discussion, we developed Hypothesis 2:

Hypothesis 2: Social identity positively influences participation behavior in cultural and creative product virtual communities.

Prior research on social identity has revealed that identification with a group produces positive outcomes, in such a way that people who strongly identify themselves with organizations are more likely to support these organizations in various ways and to evaluate these organizations positively (Ahearne et al., 2005). Consumers who identify strongly with a company are more likely to actively engage in additional behaviors, such as passing on positive WOM information about the company and influencing other customers (Castro et al., 2022). On the basis of this discussion, we developed Hypothesis 3:

Hypothesis 3: Social identity positively influences influencer behavior in cultural and creative product virtual communities.

Formerly, individuals tended to rely on social relationships and interactions with peers when exhibiting purchasing behaviors (Vivek et al., 2014). In modern market development conditions, many products are purchased by customers not because of their physical characteristics, but because the customers belong to a particular social group (Ivanova et al., 2022). In the context of online communities, social relationships are generated through the interaction of members in the community. On the basis of this discussion, we developed Hypothesis 4:

Hypothesis 4: Social identity positively influences purchase behavior in cultural and creative product virtual communities.

Customer influencer behavior can influence customers’ perceptions. This study views WOM in communities as the perceived behavior of community members having an influence on others, which represents influencer behavior in this paper. In many product categories, WOM, interaction, and assistance from other customers post-acquisition can significantly affect people’s behavior through increased persuasion, the conversion of people into customers and the recipient customer’s continued usage of a product (Katz and Shapiro, 1985). Several researchers have studied the role of individuals’ influence on others in the diffusion and adoption of products (Van den Bulte and Wuyts, 2007). It is widely acknowledged that WOM can significantly influence a firm’s sales and consumer behavioral intentions (Tseng and Hsu, 2010). The worldwide spread of the Internet has engendered a less personal but more ubiquitous form of WOM communication: online WOM (e-WOM) communication (Jalilvand and Samiei, 2012). The influencer behavior represented by WOM that is explored in this paper is e-WOM behavior in the community. This new type of WOM communication has become an important venue for consumer opinions (Chih et al., 2013) and is assumed to be even more effective than in-person WOM communication due to its greater accessibility and reach (Chatterjee, 2001). Reza Jalilvand et al. (2012) argued that e-WOM is one of the most effective factors influencing brand purchase intention in consumer markets. Investigations, have found that e-WOM information impacts purchase intention. Thus, we have defined connections between the constructs of influencer behavior and purchase intention, and have generated Hypothesis 5:

Hypothesis 5: Influencer behavior mediates the influence of social identity on purchase intention in cultural and creative product virtual communities.

## Materials and Methods

### Sample and Data Collection

Data were collected through an online self-reported survey supported by Sojump, a questionnaire website that is widely used in mainland China. The study used convenience sampling, which is a type of non-probability sampling. Because the research scope was limited to online communities, we choose to distribute our questionnaire in online virtual communities. In addition, because this study examines cultural and creative product users, the virtual communities in which we chose to distribute the questionnaire were related to cultural and creative products, where the users’ interests were consistent with the nature of the communities. The questionnaires were distributed in virtual communities based in the Chinese mainland (e.g., Douban groups, Weibo communities, and WeChat groups). This questionnaire was developed on the basis of evidence from prior literature on social identity theory and CE. Of the questionnaires distributed, 661 were returned. After removing invalid questionnaires, we obtained 520 samples, yielding a 78.7% response rate from the participants.

According to Chinese investigative reports on Chinese cultural and creative products in 2019 (PR World, 2019), more than 1 billion online consumers of cultural and creative products were born during the period 1990–1999, which is consistent with our data. The generation born during this decade is known as Generation Z. Unlike previous generations, Generation Z are Internet natives, who are more reliant on digital media and more active within online communities. They are also savvy consumers who do not trust brands and prefer personalized services (Gutfreund, 2016). They trust information shared with their peers within spontaneously engaged online communities more than information that they receive through direct advertising. This is in line with the demographic profile of respondents as belonging to Generation Z. Furthermore, our gender data is also in line with the aforementioned investigative reports. The demographic information of the participants is presented in Table 1.

**TABLE 1:** Demographic profile of the participants.

Variable	Characteristic	Frequency	Percentage
Gender	Male	210	40.38
	Female	310	59.62
	Total	520	100.0
Age	Born after 2000	47	9.04
	Born 1990–1999	403	77.50
	Born 1980–1989	47	9.04
	Born 1970–1979	23	4.42
	Total	520	100.0

### Measures

The scale of measurement for the constructs included in the research model was defined on the basis of the measures used in the sources, as shown in Table 2.

**TABLE 2:** Measurement items.

Constructs	Items	References
Cognitive social identity	csi1	Wang, 2017
	csi2	
	csi3	
Affective social identity	asi1	Wang, 2017
	asi2	
	asi3	
Evaluative social identity	esi1	Wang, 2017
	esi2	
	esi3	
Knowledge behavior	kbh1	Shi, 2010
	kbh2	
	kbh3	
	kbh4	
Influencer behavior	ibh1	Jalilvand and Samiei, 2012
	ibh2	
	ibh3	
	ibh4	
	ibh5	
	ibh6	
Participation behavior	pab1	Wang, 2017
	pab2	
	pab3	
Purchase intention	pb1	Jalilvand and Samiei, 2012
	pb2	
	pb3	
	pb4	

The research model employed in this study examines the effects of cognitive, affective, and evaluative identification on CE behaviors. The questionnaire was composed of two sections. In the first section, participants indicated their agreement or disagreement with 24 items using a 7-point Likert scale that ranged from “strongly disagree” (1) to “strongly agree” (7). The advantage of using an interval scale is that it permits the researchers to use a variety of statistical techniques that can be applied to nominal and ordinal scale data, in addition to the arithmetic mean, standard deviation, product-moment correlations, and other statistics commonly used in marketing research (Malhotra and Galletta, 1999).

The 24 items were grouped into the seven constructs considered in the model. According to Ellemers et al. (1999), social identity has been commonly measured and treated as a unidimensional construct. Therefore, social identity was set as a second-order construct, as mentioned above. Cognitive, affective, and evaluative social identities were used as first-order constructs and were measured by three items each, a format adapted from Bagozzi and Dholakia (2002). The cognitive dimension of social identity refers to a person’s self-categorization as a group member (Dholakia et al., 2004). Affective identification refers to the emotional attachment of a person to the group (Ellemers et al., 1999). The evaluative aspect of social identity refers to the assessment of self-worth that results from group membership (Dholakia et al., 2004).

Three aspects of social identity were both measured with items adapted from Bagozzi and Dholakia (2002) and Dholakia et al. (2004). We refer to Wang’s (2017) adaptation of the measurement items of cognitive, affective, and evaluative identification. Engagement behavior outcomes, including knowledge behavior, influencer behavior, participation behavior, and purchase intention, were operationalized by four items, six items, two items, and four items, respectively. Knowledge behavior measurement items are referenced in the Shi’s study on ability of knowledge contribution (Shi, 2010). We chose e-WOM behavior to represent influencer behavior, following the definition of Kumar et al. (2010), and measured by the items adapted from Jalilvand and Samiei’s (2012) research. We replaced referral behavior with participation behavior, which measured with items in Wang’s (2017) research on usage of social networking site (Wang, 2017). Purchase intention measurement items are adapted from Jalilvand and Samiei’s (2012) research. All measurement items and their sources are listed in Table 2.

### Data Analysis

The quantitative data analysis included two processes: (1) an analysis of the descriptive statistics and the reliability of the measurement using SPSS 26.0 and Amos 24.0 software, and (2) structural equation modeling (SEM) testing. Amos 24.0 was used to evaluate the correlation coefficient between variables in the factor analysis and path analysis of the equation model. A detailed explanation of the application of both analytical procedures and their results is given below.

## Results

To test the validity and reliability of the measurement, confirmatory factor analysis (CFA) was conducted using AMOS 24.0. The results indicate an acceptable fit with the data (χ^2^ = 557.836, df = 163, normed χ^2^ = 3.422). The goodness of fit index (GFI = 0.899) and comparative fit index (CFI = 0.950) were also acceptable, and the standardized root mean square residual (SRMR = 0.0480) and root mean square error of approximation (RMSEA = 0.068) were less than 0.8 (HooperD and Mullen, 2008). All factor loadings were statistically significant at *p* < 0.001. The average variance extracted (AVE) for the seven constructs ranged from 0.662 to 0.826, exceeding 0.5 (Fornell and Larcker, 1981), and the composite reliability (CR) for the constructs was higher than 0.8 (Hair et al., 2009), indicating satisfactory convergent validity.

Confirmatory factor analysis revealed a standard factor loading of pab2, one of the measurements of participation behavior construct, below 0.6 (Hair et al., 2009). Thus, this measurement item was removed. To analyze one item, according to Schumacker and Lomax (2004), the CR of pab3 was set as 0.8 to modify the model, which required the use of SPSS 26.0 to calculate the covariance of item pab3 (2.734) and include it in our model. The other construct, Cronbach’s alpha, exceeded the marginal value of 0.7 (Hair et al., 2009), indicating satisfactory internal consistency for all constructs, as presented in Table 3.

**TABLE 3:** Convergent validity.

Construct		Unstd	SE	*t*-Value	*p*	Std	SMC	1-SMC	CR	AVE	Cronbach’s α
Cognitive social identity											
	CSI1	1.000				0.807	0.651	0.349	0.856	0.665	0.856
	CSI2	1.076	0.057	18.843	***	0.848	0.719	0.281			
	CSI3	0.966	0.053	18.260	***	0.791	0.626	0.374			
Affective social identity											
	ASI1	1.000				0.814	0.663	0.337	0.854	0.662	0.853
	ASI2	1.123	0.059	18.959	***	0.867	0.752	0.248			
	ASI3	0.990	0.056	17.764	***	0.757	0.573	0.427			
Evaluative social identity											
	ESI1	1.000				0.795	0.632	0.368	0.897	0.745	0.895
	ESI2	1.090	0.050	21.886	***	0.851	0.724	0.276			
	ESI3	1.217	0.053	22.981	***	0.937	0.878	0.122			
Influencer behavior											
	IB2	1.000				0.693	0.480	0.520	0.871	0.631	0.868
	IB4	1.442	0.081	17.741	***	0.886	0.785	0.215			
	IB5	1.183	0.078	15.236	***	0.731	0.534	0.466			
	IB6	1.254	0.072	17.364	***	0.852	0.726	0.274			
Knowledge behavior											
	KB2	1.000				0.864	0.746	0.254	0.878	0.706	0.878
	KB3	0.997	0.048	20.703	***	0.803	0.645	0.355			
	KB4	1.027	0.047	21.775	***	0.853	0.728	0.272			
Purchase behavior											
	PB1	1.000				0.825	0.681	0.319	0.854	0.662	0.853
	PB2	0.940	0.053	17.832	***	0.761	0.579	0.421			
	PB3	1.016	0.054	18.924	***	0.852	0.726	0.274			
Participation behavior											
	PAB3	1.501	0.056	26.756	***	0.909	0.826	0.174	0.826	0.826	0.800

Unstd, unstandardized factor loadings; Std, standardized factor loadings; SE, standard error; SMC, squared multiple correlation; CR, composite reliability; AVE, average variance extracted. ***p < 0.001.

To test discriminant validity, the values of AVE and the squared correlation of constructs were compared for each pair of constructs. All squared correlations were acceptable; thus, the discriminant validity of the constructs was satisfactory (Table 4).

**TABLE 4:** Discriminant validity.

	AVE	pabh	pbh	kbh	ibh
pabh	0.826	0.909			
pbh	0.662	0.488	0.814		
kbh	0.706	0.661	0.719	0.840	
ibh	0.631	0.286	0.853	0.595	0.794

*AVE, average variance extracted.*

According to Marsh and Hocevar (1985), by calculating the target coefficient, this study compared CFA of the first order and second order to decide the fitness with the data. At-value closer to 1 implies that the second-order CFA can replace the first-order CFA, making the model more precise. In this case, the target coefficient was used to test for the existence of a higher-order social identity construct. A target coefficient of 1 provided satisfactory evidence of a second-order social identity construct. The fitness index of the second-order CFA of social identity implies that the fitness was good.

**FIGURE 2:**
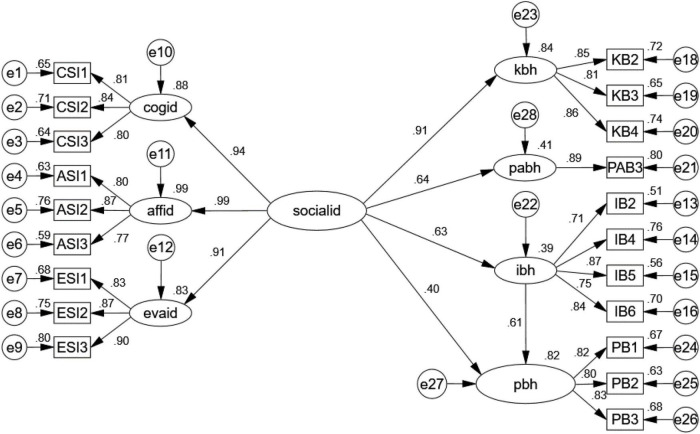
Results of the structural equation model (*N* = 520). cogid, cognitive social identity; affid, affective social identity; evaid, evaluative social identity; socialid, social identity; kbh, knowledge behavior; ibh, influencer behavior; pabh, participation behavior; pbh, purchase intention.

Structural equation modeling was conducted using AMOS 24.0 to test the hypotheses. To test the model, social identity as a second-order construct was used to test the model. As shown in Figure 2, the SEM analysis results showed that social identity had a significant influence on consumer knowledge behavior (β = 0.91, *p* < 0.001), consumer participation behavior (β = 0.64, *p* < 0.001), consumer influencer behavior (β = 0.63, *p* < 0.001), and consumer purchase intention (β = 0.40, *p* < 0.001). This result confirms that Hypotheses 1–4 were supported (Table 5).

**TABLE 5:** Hypotheses testing.

	Hypothesis	Structural loading	SE	*t*-Value	*p*	Result
H1	Social identity → knowledge behavior	0.914	0.056	19.004	***	Supported
H2	Social identity → participation behavior	0.622	0.058	14.965	***	Supported
H3	Social identity → influencer behavior	0.625	0.045	12.936	***	Supported
H4	Social identity → purchase intention	0.395	0.044	8.291	***	Supported

***p < 0.001.

To test Hypothesis 5, which explored whether influencer behavior has a mediating effect on the relationship between social identity and purchase behavior, we conducted bias-corrected percentile bootstrapping and percentile bootstrapping at a 95% confidence interval with 1,000 bootstrap samples (Taylor et al., 2008). We also calculated the lower and upper bounds of the confidence interval to test for indirect effects. As presented in Table 6, the results for the test of indirect effects from social identity to purchase intentions, where it can be seen that effects exist (indirect effect *Z* = 7.72 > 1.96) (Sobel, 1982). The results confirm that Hypothesis 5 was supported.

**TABLE 6:** Unstandardized direct, indirect, and total effects of the hypothesized model.

		Point estimate	Product of coefficients		Bootstrapping				Two-tailed significance
					Bias-corrected 95% CI		Percentile 95% CI		
			SE	*Z*	Lower	Upper	Lower	Upper	
Total effects	SI → pb	0.788	0.059	13.356	0.680	0.906	0.683	0.912	0.00**
Indirect effects	SI → pb	0.386	0.050	7.720	0.303	0.502	0.299	0.499	0.00**
Direct effects	SI → pb	0.402	0.060	6.700	0.285	0.529	0.287	0.531	0.00**

Unstandardized estimation of 1,000 bootstrap samples, **p < 0.01.

**FIGURE 3:**
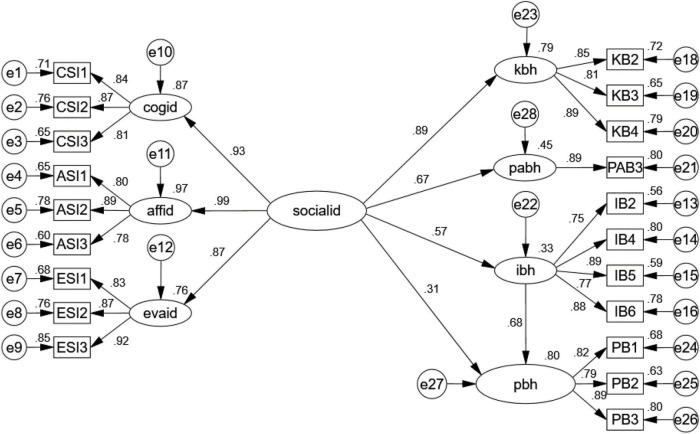
Results of the structural equation model with the female sample. χ^2^ = 487.649, df = 163, normed χ^2^ = 2.992, goodness of fit index (GFI) = 0.860, AGFI = 0.819, comparative fit index (CFI) = 0.936, standardized root mean square residual (SRMR) = 0.058, root mean square error of approximation (RMSEA) = 0.08.

**FIGURE 4:**
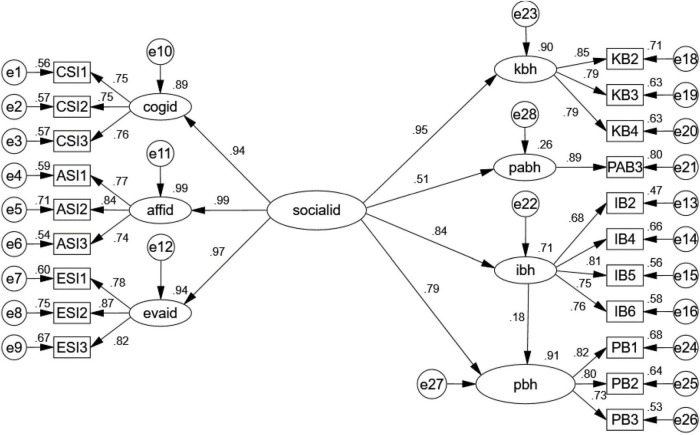
Results of the structural equation model with the male sample. χ^2^ = 331.564, df = 163, normed χ2 = 2.034, goodness of fit index (GFI) = 0.864, AGFI = 0.825, comparative fit index (CFI) = 0.941, standardized root mean square residual (SRMR) = 0.0476, root mean square error of approximation (RMSEA) = 0.07.

According to the Chinese investigation reports of Chinese cultural and creative products in 2019 (PR World, 2019), women had stronger intentions to purchase the products. Based on this report, we aimed to examine whether differences existed between our model’s samples of men and women. Thus, we conducted a multi-sample analysis. Multi-sample analysis with unconstrained model showed the model fit the data well [χ^2^ (326) = 819.22, *p* < 0.05, NFI = 0.90, NNFI = 0.93, CFI = 0.94, RMSEA = 0.05]. Then, to test the invariance of the factor loadings across gender, we constrained factor loadings to be equal across two groups. Data showed constrained model fit well [χ^2^ (339) = 838.82, *p* < 0.05, NFI = 0.90, NNFI = 0.93, CFI = 0.94, RMSEA = 0.05]. Also, the χ^2^ test between unconstrained and constrained model was not significant [χ^2^ (13) = 19.607, *p* = 0.11 > 0.05], showing that factor loadings of both gender groups were invariant (Cheung and Rensvold, 1999). Due to participation behavior is measured by on item, according to Schumacker and Lomax (2004), the CR of pab3 was set as 0.8 to modify the model. Therefore, we measured the gender factor separately after. The results of the SEM analysis of the different genders are displayed in Figures 3, 4.

According to Figures 3, 4, Hypotheses 1–4 are still supported. However, in the model with the male sample, influencer behavior doesn’t mediate the effect of social identity on purchase intention (indirect effect *Z* = 1.122 < 1.96, *p* = 0.266) (Sobel, 1982), as shown in Table 7.

**TABLE 7:** Unstandardized direct, indirect, and total effects of the female and male model.

Model		Point estimate	Product of coefficients		Bootstrapping				Two-tailed significance
					Bias-corrected 95% CI		Percentile 95% CI		
			SE	*Z*	Lower	Upper	Lower	Upper	
**Total effects**									
Model 1	SI → pb	0.668	0.065	10.277	0.547	0.793	0.544	0.790	0.00**
Model 2	SI → pb	1.126	0.137	8.219	0.893	1.450	0.892	1.450	0.00**
**Indirect effects**									
Model 1	SI → pb	0.370	0.055	6.727	0.270	0.496	0.263	0.481	0.00**
Model 2	SI → pb	0.184	0.164	1.122	−0.110	0.493	−0.096	0.514	0.266
**Direct effects**									
Model 1	SI → pb	0.297	0.060	4.950	0.187	0.420	0.183	0.417	0.00**
Model 2	SI → pb	0.941	0.203	4.635	0.556	1.377	0.537	1.348	0.00**

Unstandardized estimating of 1,000 bootstrap samples, **p < 0.01; Model 1: model with the female sample; Model 2: model with the male sample.

## Discussion

In this study, we explore the direct impact of social identity generated from user-created interested community on CE to parse out the differences in effects between communities with different attributes, providing a novel perspective on the relationship of community scope to CE for future researchers to build upon and insights on the self-driven proactive psychology of consumers that managers can use to improve brand value. In addition, we explore the different effects of gender, offering a novel influencing factor between the relation of social identity and CE behaviors for the research model. The discrepancy in the results implies that gender as a variable requires further study.

We aimed to apply social identity theory to the formation of an explanation of CE behaviors in virtual communities. The nature of cultural and creative products is to some degree uneconomic, according to Chinese common sense. Thus, it seems paradoxical that community members are willing to engage in contributing their knowledge and suggestions to such a community voluntarily. Social identity theory, from the perspective of individual-based psychology, offers insights into this practice. By going beyond Zhou and Lu (2009) model, we developed a further step for cultural and creative consumer behaviors which includes both transactional and non-transactional aspects and the relationship between them. Unlike previous cultural and creative product studies which have focused on product features, this research explored the significance of personal traits. Knowledge behavior consists of various knowledge interactions, including sharing feedback and suggestions in virtual communities. These spontaneous actions tend to be unprofitable for consumers and require self-motivation, which results from social identity in the community. In this process, cognitive, affective, and evaluative identities have almost equal impacts on social identity, as this research verifies. Interaction frequency in the cultural and creative online community, which is a measurement of participation behavior, represents the degree to which someone wants to invest time in that community even though they have multiple choices in the virtual world. The study broadens perspectives on cultural and creative products by incorporating social identity theory and CE behavior, not only considering personal traits as antecedents but also examining the interconnections between non-transactional behavior and purchasing behavior. Previous literature in the social network context has been more centered on brand fan pages (Kucharska, 2017; Chen and Tsai, 2020) rather than virtual communities. The target groups of this study were selected from several social network communities in the Chinese mainland. The concept of social identity was developed based on these virtual communities, further expanding the context of social networks.

The participants in our research came from both interest-based virtual communities (e.g., Douban and Weibo) and small-group-based virtual communities (WeChat). The interest-based community was comprised of people who shared the same interests in cultural and creative products and who shared content about their purchasing experiences. Long-term investment in the operation of brand pages while ignoring the value of virtual communities may no longer be a good strategy. The role of social identity in virtual communities must be taken into account by firms, and it is possible to do this by making only slight strategic changes. Firms can actively foster customer relationships in communities by posting content and interacting with consumers, allowing the firms to indirectly increase transactions and reduce transactional costs. The more that firms connect with influencers, the more WOM information transmission they can achieve. A previous study has proven that influencer behaviors play crucial roles in purchase behavior as well as in community activity (Wielki, 2020). Therefore, firms benefit from maintaining healthy relationships with influential supporters. A favorable community atmosphere is the result of the maintenance of a group, which provides a spirit of connection between people and enhances members’ affective, cognitive, and evaluative experiences. Some studies have examined whether companies should strategically cultivate environments that encourage customers to assist other customers (Verhoef et al., 2010). In this case, a firm may need to balance its responsibilities as both organizer and participant. In addition, people like to contribute content and information in a positive community atmosphere, and therefore positive feedback is repeatedly activated. It is easy to understand that a positive atmosphere leads to customer recognition. Taking cultural and creative firms as an example, the more that attractive content is shared with in-group members, the more the members are likely to reply and share similar items in the community. At the same time, companies can use enthusiastic users as a tool to gain new customers and increase sales. Community value is reflected in the recognition of members on both cognitive and evaluative levels, which is based on rational judgment. On an affective level, people’s need to belong can be met in a virtual community by engaging with others who have similar values. Firms and communities are not mutually exclusive, as the people that firms serve have double identities as consumers and community members. To achieve the common goal of creating people’s social identity to stimulate CE behavior, it is crucial to consider how consumers think.

The effect of social identity on customer behavior can be explained by the standardized regression coefficient. After noticing that female customers are more prone to influencing purchasing behaviors, we split our sample into two groups to examine whether gender affects the relationships between model constructs and to explore how social identity affects female and male customers’ behaviors separately. This analysis provided us with some interesting insights. Social identity is positively and significantly related to knowledge behavior for both male and female consumers. We found a positive impact of social identity on influencer behavior that was significantly stronger for men (β = 0.84, *p* < 0.001) than for women (β = 0.57, *p* < 0.001). Conversely, the effect of social identity on participation behavior was slightly greater for women (β = 0.67, *p* < 0.001) than for men (β = 0.51, *p* < 0.001). In addition, influencer behavior had a significant mediating effect on social identity and purchase behavior only in the female model. Not surprisingly, these results support prior studies. However, our study went a step further, to find that gender-based discrepancies could explain to some degree why female customers have higher purchasing intentions for cultural and creative products. The results further imply that the more that influencer behavior exists in virtual communities, the more women will participate in transaction behaviors. Previous studies have characterized men as agentic (assertive, self-centered) and women as communal (friendly, other-oriented) (Putrevu, 2001). Women can easily sympathize with others’ opinions and interests when they connect with others (Putrevu, 2001), which may partially explain why influencer behavior mediates the effect of social identity on purchase behavior in the female model. In the process of online shopping, the identification of groups is somewhat significant for women and can prompt their interaction with products.

## Limitations and Future Research

In their study of virtual community, Dholakia et al. (2004) categorized virtual community types as either network-based or small-group-based. In our research, we focused on the effects of social identity on customer behaviors. Future studies may be conducted on the features of different types of virtual communities. There are several types of Chinese mainland virtual communities, including network-based communities where public discussions are open to anyone, and small-group-based communities where discussions are private and for members only. Furthermore, the comment areas below online videos have gradually become a type of knowledge and information exchange community where followers gather. Moreover, the relationship between how firms get involved in virtual communities and customers’ engagement behaviors has not yet been explored, and should be discussed in depth in the future.

## Data Availability Statement

The datasets presented in this study can be found in online repositories. The names of the repository/repositories and accession number(s) can be found in the article/Supplementary Material.

## Ethics Statement

The studies involving human participants were reviewed and approved by the University of Shanghai for Science and Technology Ethics Committee. The patients/participants provided their written informed consent to participate in this study.

## Author Contributions

ZZ and WL: drafting of the manuscript, acquisition of data, and statistical analysis. Both authors contributed to the article and approved the submitted version.

## Conflict of Interest

The authors declare that the research was conducted in the absence of any commercial or financial relationships that could be construed as a potential conflict of interest.

## Publisher’s Note

All claims expressed in this article are solely those of the authors and do not necessarily represent those of their affiliated organizations, or those of the publisher, the editors and the reviewers. Any product that may be evaluated in this article, or claim that may be made by its manufacturer, is not guaranteed or endorsed by the publisher.
