# Supplementary material for: Customer Engagement Around Cultural and Creative Products: The Role of Social Identity
Source: Front Psychol. 2022 Apr 25;13:874851. doi: 10.3389/fpsyg.2022.874851 (PMC9083115; doi:10.3389/fpsyg.2022.874851)
Supplement: Supplementary file 1 [file Table_1.docx]

| rowtype | varname | PB3 | PB2 | PB1 | PAB3 | KB4 | KB3 | KB2 | IB6 | IB5 | IB4 | IB2 | ESI3 | ESI2 | ESI1 | ASI3 | ASI2 | ASI1 | CSI3 | CSI2 | CSI1 |
| --- | --- | --- | --- | --- | --- | --- | --- | --- | --- | --- | --- | --- | --- | --- | --- | --- | --- | --- | --- | --- | --- |
| cov | PB3 | 2.266 |  |  |  |  |  |  |  |  |  |  |  |  |  |  |  |  |  |  |  |
| cov | PB2 | 1.545 | 2.433 |  |  |  |  |  |  |  |  |  |  |  |  |  |  |  |  |  |  |
| cov | PB1 | 1.56 | 1.554 | 2.334 |  |  |  |  |  |  |  |  |  |  |  |  |  |  |  |  |  |
| cov | PAB3 | 0.906 | 0.903 | 0.912 | 2.711 |  |  |  |  |  |  |  |  |  |  |  |  |  |  |  |  |
| cov | KB4 | 1.327 | 1.322 | 1.335 | 1.289 | 3.04 |  |  |  |  |  |  |  |  |  |  |  |  |  |  |  |
| cov | KB3 | 1.279 | 1.275 | 1.287 | 1.243 | 2.183 | 3.23 |  |  |  |  |  |  |  |  |  |  |  |  |  |  |
| cov | KB2 | 1.252 | 1.247 | 1.259 | 1.216 | 2.136 | 2.06 | 2.8 |  |  |  |  |  |  |  |  |  |  |  |  |  |
| cov | IB6 | 1.336 | 1.331 | 1.344 | 0.739 | 1.082 | 1.043 | 1.021 | 2.248 |  |  |  |  |  |  |  |  |  |  |  |  |
| cov | IB5 | 1.311 | 1.307 | 1.319 | 0.726 | 1.062 | 1.024 | 1.002 | 1.557 | 2.706 |  |  |  |  |  |  |  |  |  |  |  |
| cov | IB4 | 1.537 | 1.532 | 1.546 | 0.851 | 1.245 | 1.2 | 1.175 | 1.825 | 1.791 | 2.744 |  |  |  |  |  |  |  |  |  |  |
| cov | IB2 | 1.113 | 1.109 | 1.119 | 0.616 | 0.901 | 0.869 | 0.85 | 1.321 | 1.297 | 1.52 | 2.138 |  |  |  |  |  |  |  |  |  |
| cov | ESI3 | 1.49 | 1.485 | 1.499 | 1.448 | 2.119 | 2.043 | 2 | 1.215 | 1.193 | 1.398 | 1.012 | 3.606 |  |  |  |  |  |  |  |  |
| cov | ESI2 | 1.434 | 1.429 | 1.442 | 1.393 | 2.039 | 1.966 | 1.924 | 1.17 | 1.148 | 1.346 | 0.974 | 2.779 | 3.538 |  |  |  |  |  |  |  |
| cov | ESI1 | 1.332 | 1.327 | 1.34 | 1.294 | 1.894 | 1.826 | 1.787 | 1.086 | 1.066 | 1.25 | 0.905 | 2.581 | 2.484 | 3.395 |  |  |  |  |  |  |
| cov | ASI3 | 1.273 | 1.269 | 1.281 | 1.237 | 1.811 | 1.745 | 1.708 | 1.038 | 1.019 | 1.195 | 0.865 | 2.033 | 1.957 | 1.818 | 2.973 |  |  |  |  |  |
| cov | ASI2 | 1.432 | 1.427 | 1.441 | 1.392 | 2.037 | 1.964 | 1.922 | 1.168 | 1.147 | 1.344 | 0.973 | 2.288 | 2.202 | 2.045 | 1.976 | 2.919 |  |  |  |  |
| cov | ASI1 | 1.24 | 1.236 | 1.248 | 1.205 | 1.764 | 1.7 | 1.664 | 1.012 | 0.993 | 1.164 | 0.843 | 1.981 | 1.906 | 1.771 | 1.711 | 1.925 | 2.627 |  |  |  |
| cov | CSI3 | 1.156 | 1.152 | 1.163 | 1.123 | 1.644 | 1.585 | 1.551 | 0.943 | 0.925 | 1.085 | 0.785 | 1.846 | 1.777 | 1.65 | 1.577 | 1.774 | 1.537 | 2.541 |  |  |
| cov | CSI2 | 1.258 | 1.254 | 1.266 | 1.223 | 1.789 | 1.725 | 1.688 | 1.026 | 1.007 | 1.181 | 0.855 | 2.01 | 1.934 | 1.796 | 1.717 | 1.932 | 1.673 | 1.768 | 2.734 |  |
| cov | CSI1 | 1.189 | 1.185 | 1.196 | 1.155 | 1.691 | 1.63 | 1.596 | 0.97 | 0.952 | 1.116 | 0.808 | 1.899 | 1.828 | 1.698 | 1.623 | 1.826 | 1.581 | 1.671 | 1.819 | 2.615 |
| n |  | 520 | 520 | 520 | 520 | 520 | 520 | 520 | 520 | 520 | 520 | 520 | 520 | 520 | 520 | 520 | 520 | 520 | 520 | 520 | 520 |

Appendix

Table 1. Sample Covariance Matrix
